# Supplementary material for: Fragile neutrophils in surgical patients: A phenomenon associated with critical illness
Source: PLoS One. 2020 Aug 4;15(8):e0236596. doi: 10.1371/journal.pone.0236596 (PMC7402494; doi:10.1371/journal.pone.0236596)
Supplement: S1 Fig — (A) White cell viability fraction in polytrauma patients over days after trauma. (B) White cell viability fraction in polytrauma patients who develop organ dysfunction relative to the first day organ dysfunction became clinically evident. Patients with organ dysfunction (n = 11) are depicted in red (▲) and patients without organ dysfunction (n = 70) are depicted in green (●). Organ dysfunction is defined as acute respiratory distress syndrome and/or multiple organ dysfunction syndrome. In Fig 1B, day 0 is the first day that organ dysfunction became clinically evident. Data are presented as mean with standard error of the mean (SEM). (PDF) [file pone.0236596.s001.pdf]

S1 Fig. White cell viability fraction in polytrauma patients developing organ dysfunction.

A

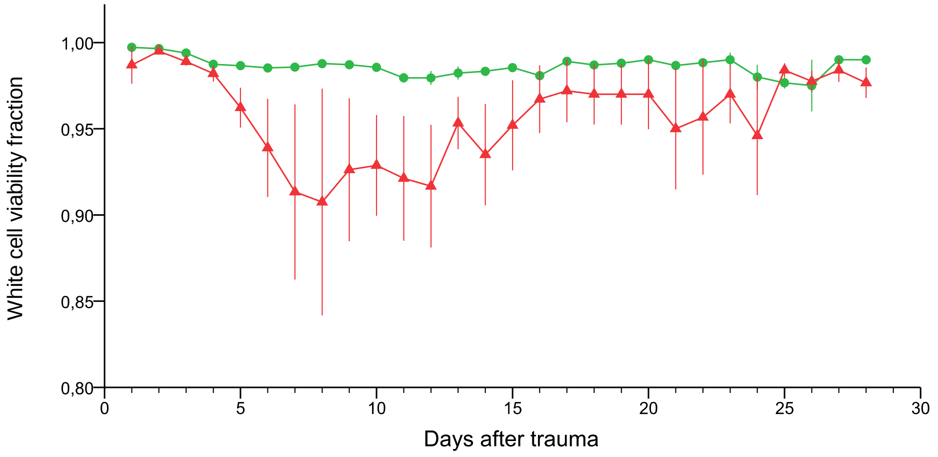

B

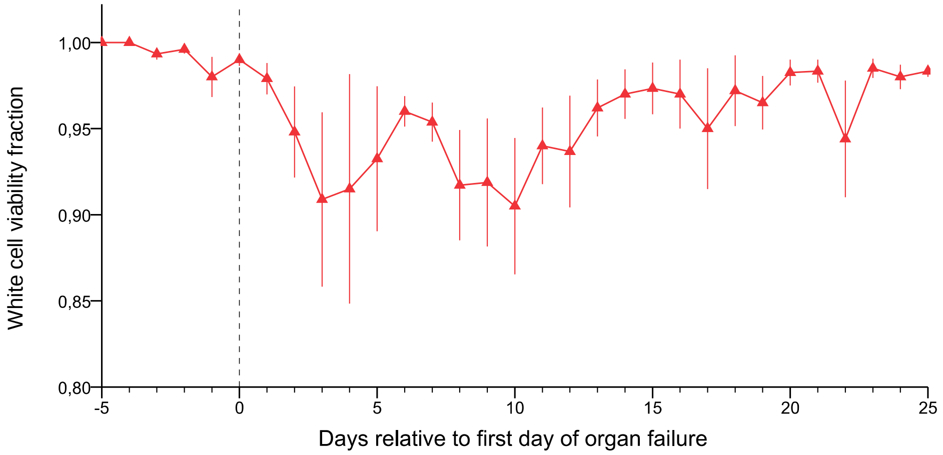

(A) White cell viability fraction in polytrauma patients over days after trauma. (B) White cell viability fraction in polytrauma patients who develop organ dysfunction relative to the first day organ dysfunction became clinically evident. Patients with organ dysfunction (n = 11) are depicted in red (▲) and patients without organ dysfunction (n = 70) are depicted in green (●). Organ dysfunction is defined as acute respiratory distress syndrome and/or multiple organ dysfunction syndrome. In Figure 1B, day 0 is the first day that organ dysfunction became clinically evident. Data are presented as mean with standard error of the mean (SEM).
