# Supplementary material for: Fragile neutrophils in surgical patients: A phenomenon associated with critical illness
Source: PLoS One. 2020 Aug 4;15(8):e0236596. doi: 10.1371/journal.pone.0236596 (PMC7402494; doi:10.1371/journal.pone.0236596)
Supplement: S2 Fig — Leukocyte count (●) and non-viable leukocyte count (▲) over time in patients with fragile neutrophils (n = 9). Day 0 represents the first day that the white cell viability fraction was ≤ 0.95 (dotted line). Leukocyte numbers decreased before the number of non-viable leukocytes increased. Still, leukocyte counts were above reference values (reference range adults: 4.0–11 x 109 / L) for almost the entire period. Data are presented as mean with standard error of the mean. (PDF) [file pone.0236596.s002.pdf]

S2 Fig. Total leukocyte count and non-viable leukocyte count over time in patients with fragile neutrophils.

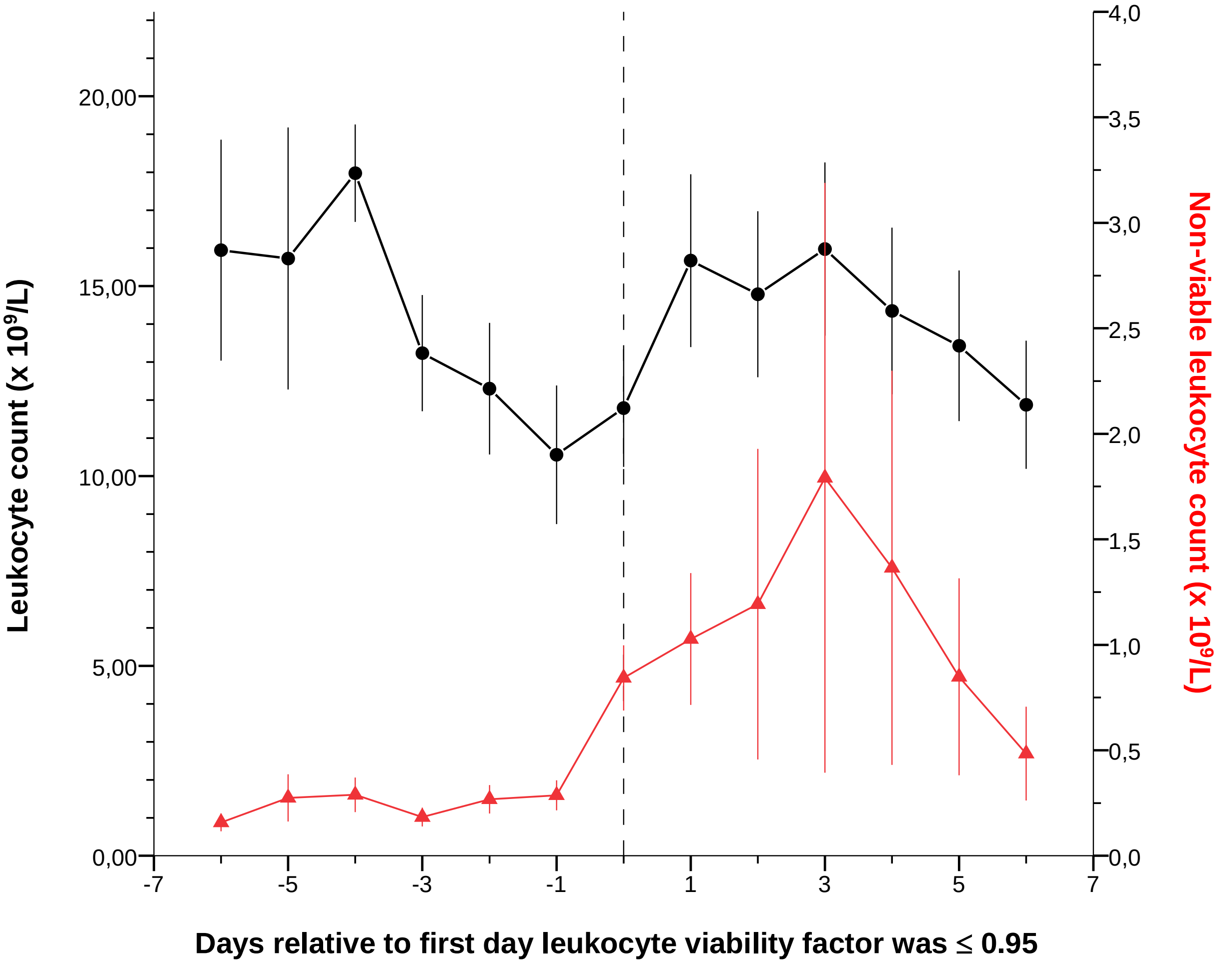

Leukocyte count (●) and non-viable leukocyte count (▲) over time in patients with fragile neutrophils (n = 9). Day 0 represents the first day that the white cell viability fraction was ≤ 0.95 (dotted line). Leukocyte numbers decreased before the number of non-viable leukocytes increase. Still, leukocyte counts were above reference values (reference range adults: 4.0 – 11 x 10^9 / L) for almost the entire period. Data are presented as mean with standard error of the mean.
