# Supplementary figures and images for: Fragile neutrophils in surgical patients: A phenomenon associated with critical illness
Source: PLoS One. 2020 Aug 4;15(8):e0236596. doi: 10.1371/journal.pone.0236596 (PMC7402494; doi:10.1371/journal.pone.0236596)

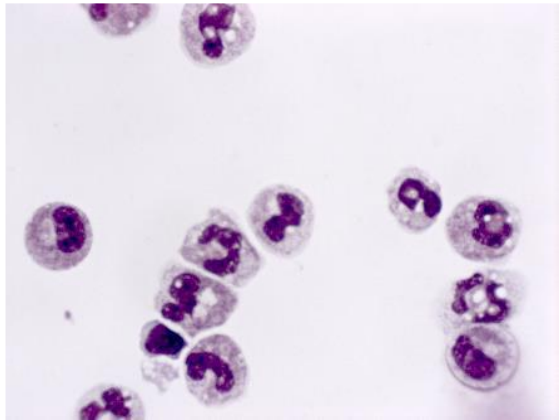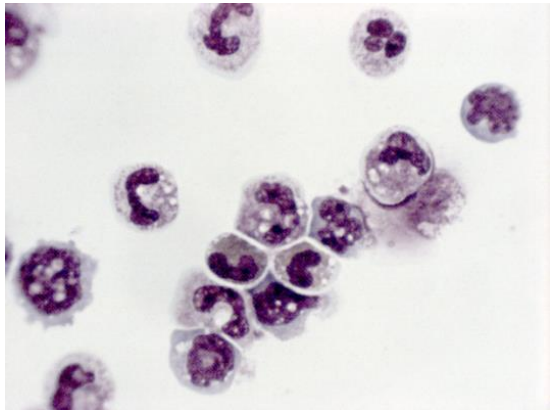

Supplement: S3 Fig — Leukocyte cytospins of patients with fragile neutrophils revealed remarkable morphological neutrophil characteristics that are associated with severe inflammation. Firstly, banded neutrophils and other progenitors were often observed in these cytospins. Secondly, some cytospins contained neutrophils with cytoplasmic vacuoles and toxic granulation. (PDF) [file pone.0236596.s003.pdf]

**A**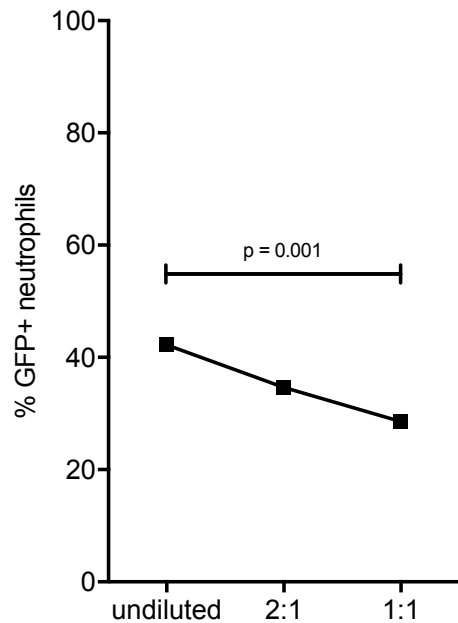**B**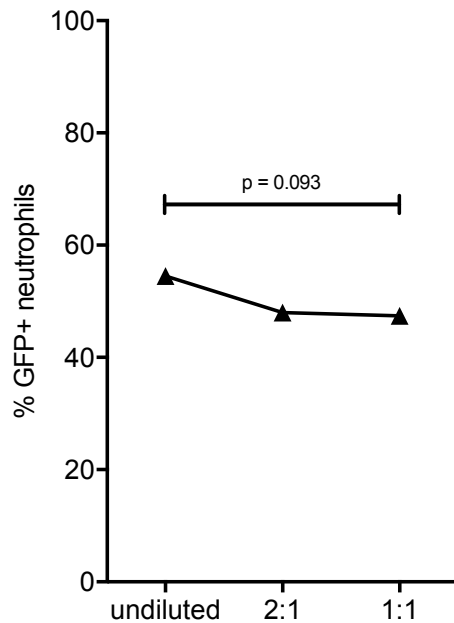

Supplement: S4 Fig — (A) Percentage of GFP-positive neutrophils in different dilutions measured after 20 minutes of incubation with S. Aureus-GFP. (B) Percentage of GFP-positive neutrophils in different dilutions measured after 40 minutes of incubation with S. Aureus-GFP. Whole blood samples of healthy controls (n = 5) were diluted with plasma obtained from the same sample by centrifugation. The following whole blood (WB) to plasma (P) ratios were used: 1WB:0P (undiluted), 2WB:1P, 1WB:1P. Than S. Aureus-GFP was added to all samples with a MOI of 1 (bacteria to phagocytes ratio of 1:1). The whole blood with bacteria was shaken at 37°C for 40 minutes. After 20 and 40 minutes, part of the sample was removed and put on ice. Leukocytes were stained for 15 minutes on ice with CD45-APC for recognition during analysis. After staining, cells were fixed with paraformaldehyde (PFA) 1% and the percentage of GFP-positive neutrophils was measured on the BD LSRFortessa™. Data are presented as mean. The percentage of GFP-positive neutrophils was compared between samples with different dilutions using the Friedman test for paired data. Dilution of whole blood led to a decrease in the percentage of GFP-positive neutrophils. This decrease was significant in samples analyzed after 20 minutes of incubation (p = 0.001). GFP = Green fluorescent protein. MOI = multiplicity of infection. S. Aureus = Staphylococcus Aureus. (PDF) [file pone.0236596.s004.pdf]
