# Supplementary material for: Fragile neutrophils in surgical patients: A phenomenon associated with critical illness
Source: PLoS One. 2020 Aug 4;15(8):e0236596. doi: 10.1371/journal.pone.0236596 (PMC7402494; doi:10.1371/journal.pone.0236596)
Supplement: S1 Table — (DOCX) [file pone.0236596.s005.docx]

**S1 Table. Correlation between neutrophil marker expression and white cell viability fraction.**

| **WVF vs.** | **Pearson correlation, R** | **P-value** |
| --- | --- | --- |
| CD35 | 0.432 | 0.246 |
| CD66b | 0.522 | 0.150 |
| CD64 | -0.038 | 0.922 |
| CBRM1/5 | 0.364 | 0.336 |
| CD11b | 0.521 | 0.150 |
| CD14 | -0.016 | 0.967 |
| CD16 | 0.278 | 0.469 |
| CD62L | -0.163 | 0.675 |
| LAIR1 | 0.184 | 0.636 |
| CD49d | 0.559 | 0.249 |
