# Supplementary material for: Fragile neutrophils in surgical patients: A phenomenon associated with critical illness
Source: PLoS One. 2020 Aug 4;15(8):e0236596. doi: 10.1371/journal.pone.0236596 (PMC7402494; doi:10.1371/journal.pone.0236596)
Supplement: S1 File — (PDF) [file pone.0236596.s006.pdf]

# Neutrophil viability in surgical patients and its relationship with neutrophil functionality

*Neutrofiel levensvatbaarheid in chirurgische patiënten en de relatie met neutrofiel functionaliteit*

**PROTOCOL TITLE "**Neutrophil viability in surgical patients and its relationship with neutrophil functionality"

| | |
|---|---|
| **Protocol ID** | |
| **Short title** | **Neutrophil viability study** |
| **Version** | **4** |
| **Date** | **20-03-2018** |
| **Project leader** | **Prof. Dr. L.P.H Leenen**<br>**Department of Trauma Surgery**<br>**University Medical Center Utrecht. HP G.04.228**<br>**PO Box 85500, 3508 GA Utrecht**<br>**Phone: +31 88 755 9882**<br>**l.p.h.leenen@umcutrecht.nl** |
| **Principal investigator** | **Dr. Hietbrink**<br>**Department of Trauma Surgery**<br>**University Medical Center Utrecht. HP G04.228,**<br>**PO Box 85500, 3508 GA Utrecht.**<br>**Phone: +31 8875559882**<br>**F.Hietbrink@umcutrecht.nl** |
| **Executive investigators** | **Drs. L. Hesselink**<br>**Department of Trauma surgery**<br>**University Medical Center Utrecht**<br>**l.hesselink@umcutrecht.nl**<br><br>**Drs. R. Spijkerman**<br>**Department of Trauma surgery**<br>**University Medical Center Utrecht**<br>**r.spijkerman-5@umcutrecht.nl** |
| **Other investigators** | **Drs. P Hellebrekers**<br>**Department of Trauma Surgery**<br>**University Medical Center Utrecht**<br>**P.Hellebrekers@umcutrecht.nl**<br><br>**Prof. Dr L. Koenderman**<br>**Department of Respiratory Medicine**<br>**University Medical Center Utrecht**<br>**l.koenderman@umcutrecht.nl**<br><br>**Dr. I. Hoefer**<br>**Department of Clinical Chemistry and Haematology**<br>**University Medical Center Utrecht**<br>**i.hoefer@umcutrecht.nl**<br><br>**Dr. A. Huisman**<br>**Department of Clinical Chemistry and Haematology**<br>**University Medical Center Utrecht**<br>**a.huisman@umcutrecht.nl**<br><br>**Dr. M. ten Berg**<br>**Department of Clinical Chemistry and Haematology**<br>**University Medical Center Utrecht**<br>**m.tenberg@umcutrecht.nl** |
| **Sponsor** | **University Medical Center Utrecht** |

| | |
|---|---|
| **Independent physician** | **Prof. dr. Vriens**<br>**Department of CGO**<br>**University Medical Center Utrecht**<br>**Phone: +31 88 755 8214**<br>**m.r.vriens@umcutrecht.nl** |
| **Laboratory sites** | **Laboratory of Respiratory Medicine**<br>**Prof. Dr. L. Koenderman**<br>**Department Clinical Chemistry and Haematology** |

**PROTOCOL SIGNATURE SHEET**

| Name | Signature | Date |
|---|---|---|
| **Head of Department of Trauma Surgery:**<br>*Prof. Dr. L.P.H Leenen* | | |
| **Principal Investigator:**<br>*Dr. Hietbrink* | | |

**TABLE OF CONTENTS**

1. INTRODUCTION AND RATIONALE ....................................................................
2. OBJECTIVES ....................................................................................... 10
3. STUDY DESIGN ................................................................................... 10
10
4. STUDY POPULATION ........................................................................... 10
4.1 Population (base) ................................................................................ 10
4.2 Inclusion criteria ................................................................................ 11
4.3 Exclusion criteria ................................................................................ 11
4.4 Sample size calculation ........................................................................ 111
5. METHODS ........................................................................................... 12
5.1 Study parameters/endpoints ................................................................... 12
5.1.1 Main study parameter/endpoint ............................................................ 12
5.1.2 Secondary study parameters/endpoints .................................................. 12
5.2 Study procedures ................................................................................. 12
5.3 Withdrawal of individual subjects ............................................................ 15
5.4 Replacement of individual subjects after withdrawal ................................... 15
6. SAFETY REPORTING ............................................................................ 15
6.1 Section 10 WMO event ......................................................................... 15
6.2 AEs, SAEs .......................................................................................... 16
6.2.1 Adverse events (AEs) ......................................................................... 16
6.2.2 Serious adverse events (SAEs) ............................................................. 16
6.3 Follow-up of adverse events ................................................................... 17
6.4 Data Safety Monitoring Board (DSMB) / Safety Committee .......................... 17
7. STATISTICAL ANALYSIS ....................................................................... 17
7.1 Primary study parameter ....................................................................... 17
7.2 Secondary study parameters .................................................................. 17
8. ETHICAL CONSIDERATIONS .................................................................. 18
8.1 Regulation statement ............................................................................ 18
8.2 Recruitment and consent ....................................................................... 18
8.3 Objection by minors or incapacitated subjects ........................................... 18
8.4 Benefits and risks assessment, group relatedness ....................................... 19
8.5 Compensation for injury ........................................................................ 19
8.6 Incentives ........................................................................................... 19
9. ADMINISTRATIVE ASPECTS, MONITORING AND PUBLICATION ........................ 19
9.1 Handling and storage of data and documents ............................................. 19
9.2 Monitoring and Quality Assurance ........................................................... 20
9.2.1 General ............................................................................................ 20
9.2.2 Monitoring plan ................................................................................. 20
9.3 Amendments ....................................................................................... 21
9.4 Annual progress report .......................................................................... 21
9.5 End of study report .............................................................................. 22
9.6 Public disclosure and publication policy .................................................... 22
10. REFERENCES ..................................................................................... 22

## LIST OF ABBREVIATIONS AND RELEVANT DEFINITIONS

| | |
|---|---|
| **ABR** | **ABR form, General Assessment and Registration form, is the application form that is required for submission to the accredited Ethics Committee (In Dutch, ABR = Algemene Beoordeling en Registratie)** |
| **AE** | **Adverse Event** |
| **AR** | **Adverse Reaction** |
| **CA** | **Competent Authority** |
| **CCMO** | **Central Committee on Research Involving Human Subjects; in Dutch: Centrale Commissie Mensgebonden Onderzoek** |
| **CV** | **Curriculum Vitae** |
| **fMLP** | **N-formyl-methionyl-leucyl-phenylalanine** |
| **EU** | **European Union** |
| **GCP** | **Good Clinical Practice** |
| **IB** | **Investigator's Brochure** |
| **IC** | **Informed Consent** |
| **METC** | **Medical research ethics committee (MREC); in Dutch: medisch ethische toetsing commissie (METC)** |
| **PI** | **Propidium Iodide** |
| **PMN** | **Polymorphonuclear Granulocytes or Neutrophils** |
| **(S)AE** | **(Serious) Adverse Event** |
| **Sponsor** | **The sponsor is the party that commissions the organisation or performance of the research, for example a pharmaceutical company, academic hospital, scientific organisation or investigator. A party that provides funding for a study but does not commission it is not regarded as the sponsor, but referred to as a subsidising party.** |
| **SUSAR** | **Suspected Unexpected Serious Adverse Reaction** |
| **WMO** | **Medical Research Involving Human Subjects Act (in Dutch: Wet Medisch-wetenschappelijk Onderzoek met Mensen)** |
| **VF** | **Viability factor** |

## SUMMARY

**Rationale:** Previous studies showed a decreased neutrophil viability in critically ill patients. Neutrophil viability was measured using propidium iodide (PI) staining, which visualizes diminished membrane integrity and thus cell necrosis or apoptosis. Not much is known about a decreased neutrophil viability and its implications for neutrophil functioning.

**Objective**: This study aims to validate the initial decreased viability found in the routine blood sampling and further investigate its impact on neutrophil function.

**Study design:** a prospective cohort series, diagnostic

**Study population:** Surgical patients ≥ 18 years with a leukocyte viability factor ≤ 0.95 (or 95%).

**Main study parameters/endpoints:** The primary endpoint is neutrophil cell death, determined by double staining of Annexin V and PI. Secondary endpoints are neutrophil phagocytosis and responsiveness to fMLP.

**Nature and extent of the burden and risks associated with participation, benefit and group relatedness:** A total of two 4 milliliter blood containers will be collected from the patients. Sampling this amount of blood will have a negligible influence on total blood volume. In total, one venipuncture is required to obtain the blood samples and if the patient has a central venous or peripheral arterial catheter in situ, no punctures are required.

Not much is known about neutrophil viability in critically ill patients and its implications for neutrophil functioning and infectious complications. This study aims to elucidate the significance of a decreased viability factor (VF) and its relation with other signs of cell death. In addition, it is designed to gain insights in the relation between the VF and neutrophil functionality. This information is needed to get a better understanding of the neutrophil kinetics in critically ill patients and its role in the pathogenesis of hospital acquired infections.

## 1. INTRODUCTION AND RATIONALE

Neutrophils are the hosts first line of defense for bacterial invasion and eradication. Neutropenia or neutrophil dysfunction may lead to the of development of severe bacterial infections.[1,2] Heightened neutrophil degradation might be either a cause or a sign of such an infectious state.

A classic method to show morphological changes in the dying neutrophil is propidium iodide (PI) staining. With this method diminished membrane integrity and thus cell necrosis or apoptosis can be visualized.[3] PI positive cells are also used as an indicator of a prolonged bench time of laboratory samples. The viability factor (VF) is the number of PI positive leukocytes divided by the total number of leukocytes and is expressed in the range 0-1, 1 being 100% viable. The UMC Utrecht uses the VF in all diagnostic blood samples as a quality check for timely processing of the sample.

Previous studies from our laboratory showed intriguing results concerning the viability of neutrophils. First, as an incidental finding Annexin positive neutrophils were found in patients with severe septic shock, with fatal outcome (unpublished results). Additionally, more recently an association was found between a decreased viability factor and the occurrence of organ failure (MODS and ARDS) in a polytrauma cohort (Figure 1a). These samples were analyzed within 30 minutes of withdrawal, making it unlikely that a delay in bench time was the cause of decreased viability. The viability factor started to drop at the onset of organ failure and further decreased during organ failure (Figure 1b).

**Figure 1a.** Neutrophil viability factor in polytrauma patients over time

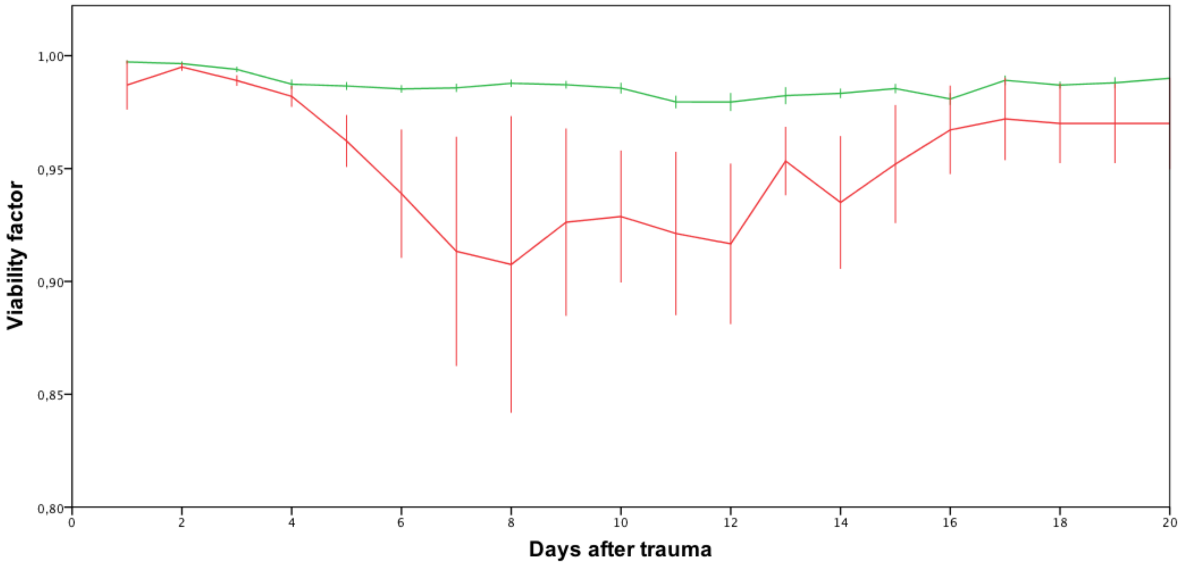

**Figure 1b.** Neutrophil viability factor in polytrauma patients relative to onset of organ failure

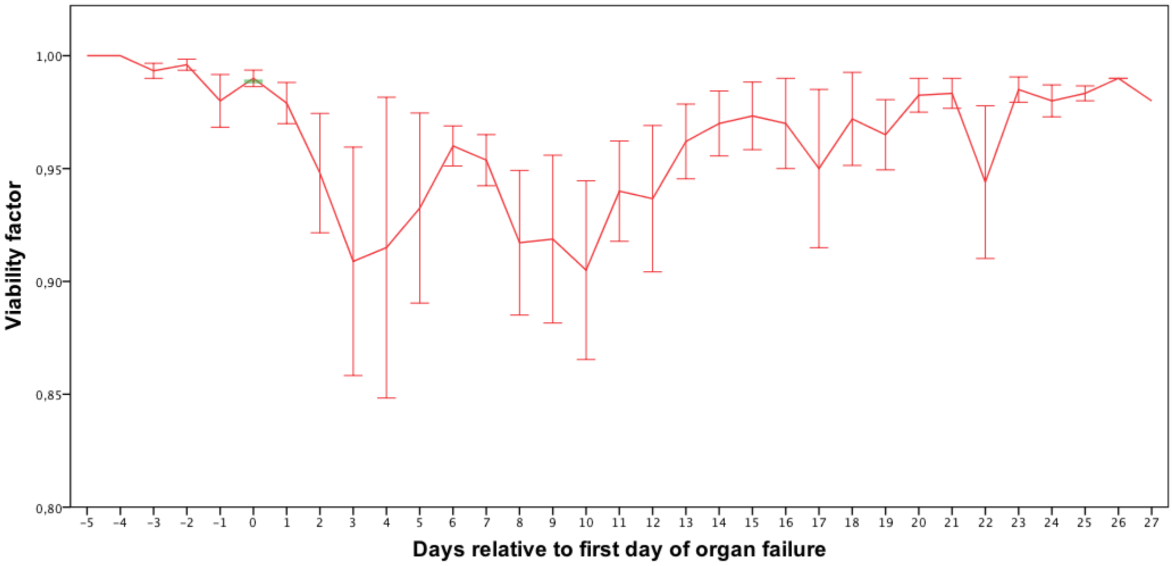


— Organ failure, — No organ failure

Organ failure is defined as ARDS or MODS.

Data are presented as mean with standard error of the mean (SEM).

We analyzed the light scatter plots of samples from trauma patients with a decreased VF (≤ 0.95). Samples with a VF ≤ 0.95, either had an increased mean PI (> 85) or a normal mean PI (< 85). Several samples with a VF ≤ 0.95 and a mean PI < 85 seemed to have a PI positive neutrophil population separate from the PI negative neutrophil population (example in figure 2a). In all samples with a VF ≤ 0.95 and a mean PI > 85 however, the whole neutrophil population seemed to have a higher PI signal (example in figure 2b). These two examples seem to depict two different phenomena that occur in critically ill trauma patients. This triggered our interest in the VF and its implications for patients with a mean PI < 85 and patients with a mean PI > 85.

**Figure 2a.** Example of sample with a VF ≤ 0.95 and a mean PI < 85

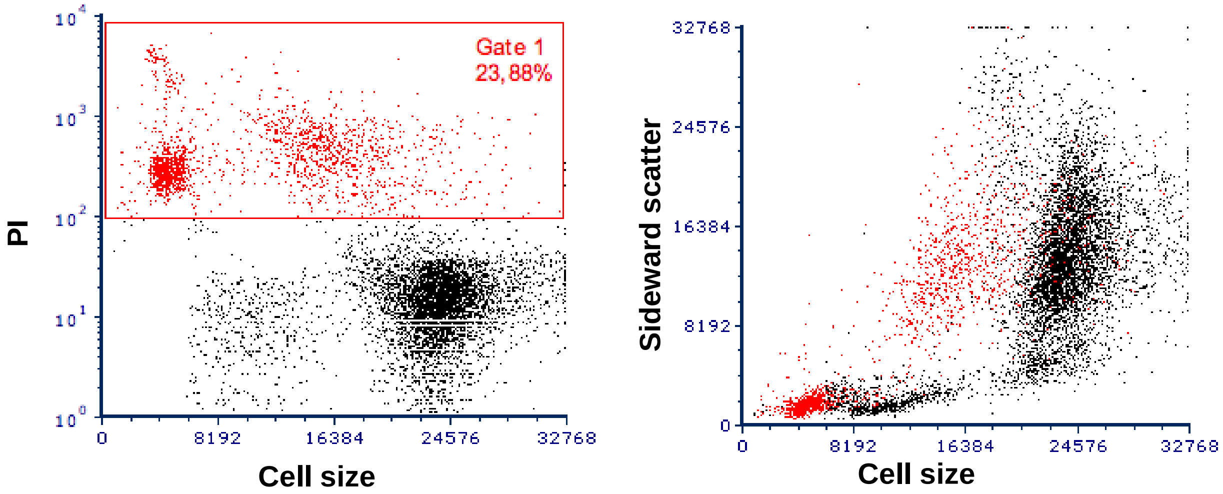

**Figure 2b.** Example of patient with a VF ≤ 0.95 and a mean PI > 85

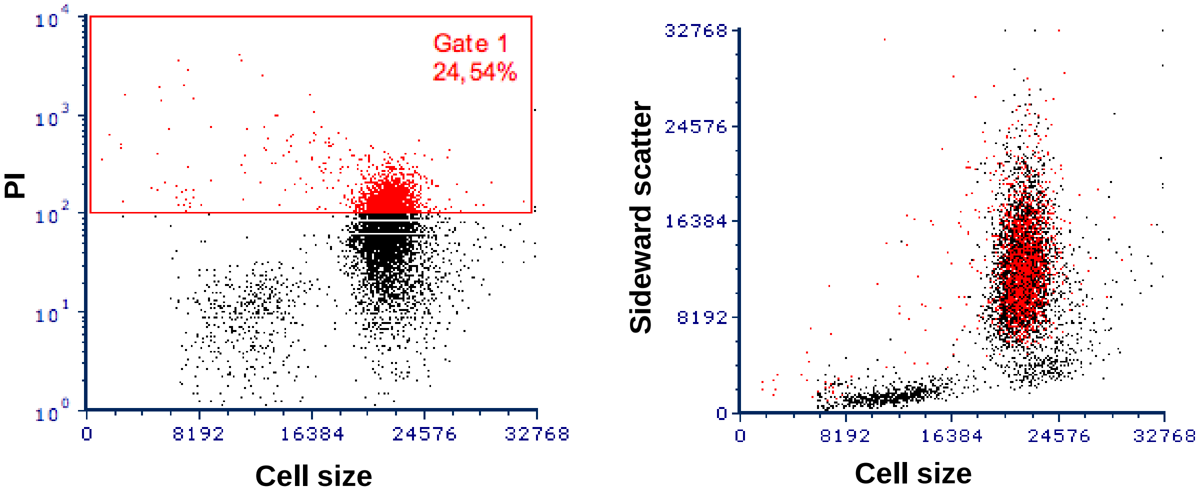


With this study we aim to determine whether VF is an adequate biomarker for neutrophil cell death. Also, the position of PI staining is analyzed (extracellular or nuclear) and associations between a decreased VF and neutrophil functionality are studied.

**Hypotheses**

1. A decreased VF in combination with a normal mean PI (< 85) *in vivo* is associated with signs of cell death in peripheral neutrophils *in vitro*.
2. A decreased VF in combination with a high mean PI (> 85) *in vivo* is associated with extranuclear DNA/RNA staining, but not with signs of cell death in peripheral neutrophils *in vitro*.

For apoptosis and necrosis assays, dual staining with Annexin V and PI is commonly used. Early apoptotic cells show PI-negative/Annexin V-positive staining, whereas late apoptotic and necrotic cells show PI-positive/Annexin V-positive staining.[4] We expect the percentage of PI positive cells to decrease after the adding of RNase, because of PI staining of RNA in the cytoplasmic compartment.[5] Also, we expect cells that are PI positive after treatment with RNase to be Annexin positive as well. We think that we will find these Annexin positive cells in samples with a decreased VF and a normal mean PI. However, we do not expect to find this in samples with a decreased VF and an increased PI, since these samples do not show a PI positive neutrophil subset separate from the PI negative neutrophils.

3. A decreased VF is associated with a decreased functionality of neutrophils

Diminished neutrophil functionality was previously found in trauma patients and patients with severe sepsis. Neutrophil degradation impairs neutrophil function and we hypothesize that a higher number of PI-positive/Annexin V positive neutrophils impairs overall neutrophil functionality. This hypothesis will be tested with a phagocytosis assay and by testing neutrophil receptor expression before and after the bacterial stimulus fMLP to test neutrophil responsiveness. We expect to find a different neutrophil receptor expression profile in samples with a VF ≤ 0.95 than in samples from healthy controls. Neutrophils from samples with a VF ≤ 0.95 are expected to have a more activated receptor profile. We think that both neutrophil phagocytosis and neutrophil responsiveness to fMLP will be impaired in patients with a decreased VF.

## 2. OBJECTIVES

*Primary Objective: :* Validate the initial decreased viability found in the routine blood sampling by determination of the actual neutrophil cell death in blood samples with a decreased VF in patients with a mean PI > 85 and patients with a mean PI < 0.85.

*Secondary Objectives*: Experimental neutrophil findings of the described study population are compared to historical data from healthy controls, provided by the '*Mini-Donor Dienst'* after given informed consent.

- Determination of the difference in neutrophil phagocytosis between surgical patients with a VF ≤ 0.95 and healthy controls *in vitro*.
- Determination of the difference in neutrophil receptor expression between surgical patients with a VF ≤ 0.95 and healthy controls *in vitro.*
- Determination of the difference in responsiveness of neutrophils to a priming stimulus with fMLP between surgical patients with a VF ≤ 0.95 and healthy controls *in vitro.*

## 3. STUDY DESIGN

A prospective cohort series, diagnostic research.

## 4. STUDY POPULATION

### 4.1 Population (base)

Surgical patients with a leukocyte viability factor ≤ 0.95 (or 95%) in routine diagnostic blood samples. Patients are recruited from the department of general surgery, trauma surgery, vascular surgery, oncologic surgery, the surgery medium care (MC) and intensive care (ICU).

A VF of .095 or lower is rare and in the UMC Utrecht only 150-200 unique patients with a decreased viability factor are seen every year. Two-thirds of all patients with a decreased viability is admitted to the intensive care unit. The patients who are not admitted to the intensive care unit, but do have a decreased viability are assumed ill and likely to be transferred to a high care ward on short term. Thus, the patient who will be allegeable for this study, will be sick and likely to have a decreased level of consciousness. To be able to include enough patients, we will have to include patient with decreased consciousness. The study population will be compared to 10 healthy controls provided by the Mini Donor Dienst (MDD) from the UMC Utrecht after informed consent is obtained.

### 4.2 Inclusion criteria

- Surgical patients
- Age ≥ 18 years
- Informed consent (when proxy consent is obtained and the patient recovers to good mental health, personal informed consent is additionally necessary)

### 4.3 Exclusion criteria

- Present or recent (<3 months) use of immunosuppressive or cytotoxic medication as defined under immunosuppressive or cytotoxic drugs by the 'Farmacotherapeutisch Kompas' (e.g. chemotoxic and cytostatic drugs. Corticosteroids, both systemically and locally used, are no exclusion criterion.)
- Use of clozapine medication since this is associated with an increased signal in the channel that measures PI staining [6]
- Known HIV positive status and related diseases

### 4.4 Sample size calculation

The aforementioned study identified 10 patients with a VF ≤ 0.95. Half of the patients had a mean PI < 85 and the other half had a mean PI > 85. In 5 patients a PI positive neutrophil population was seen separate from the PI negative neutrophil population (figure 2a). This PI positive neutrophil population was most evident in 3 of the 5 patients. Since we expect to find apoptotic or necrotic neutrophils in these samples, we want to focus primarily on these samples. To study this phenomenon at least 10 patients are needed with similar findings in their scatter plots. Hence we will include 10/3 * 10 = 33 patients. Retrospective analysis of the UPOD database demonstrated that there were 183

patients on surgical wards and the ICU with a VF ≤ 95% in 2016. Hence, the inclusion of 33 patients will take at least 2 months.

The study population will be compared to 10 healthy controls provided by the Mini Donor Dienst (MDD) from the UMC Utrecht after informed consent is obtained.

# 5. METHODS

## 5.1 Study parameters/endpoints

### 5.1.1 Main study parameter/endpoint

The primary endpoint is neutrophil cell death, determined by double staining of Annexin V and PI.

### 5.1.2 Secondary study parameters/endpoints

- Position of DNA staining in PI positive neutrophils
- Neutrophil phagocytosis
- Neutrophil receptor expression
- Neutrophil responsiveness to fMLP

## 5.2 Study procedures

*Viability alarm* - Blood samples from surgical patients are drawn as indicated by their treating physician. Every routine sample analysis, as performed by our diagnostic laboratory, contains a PI staining of leukocytes for determination of the mean PI and the VF. This is integrated in the routine test as a quality control for timely processing of blood samples. In case of prolonged bench time, the PI signal increases and VF decreases. This means that samples with decreased viability, thus increased PI signal, are flagged and the responsible laboratory technician or hematologist is alarmed. For this study population which resides within the hospital, bench time is minimal and will in most cases not be the cause for decreased viability. For the purpose of this study an additional alarm is set when the VF drops to ≤ 0.95 in a surgical patient, thereby excluding non-surgical patients. This alarm is programmed in such a way that it is directly forwarded to the PI of this study, who is the main physician or part of the team of physicians treating this patient. A notification without patient details will be send to the executive investigator. In this way, the time from

decreased viability factor to notification of the researchers is limited to a minimum (figure 3). In contrast to standard definition of a study population, in the present study a heterogeneous population consisting of surgical patients is sought to analyze the relation between the viability factor and neutrophil functionality, regardless of the patient's disease and background but with focus on the pathophysiological phenomenon.

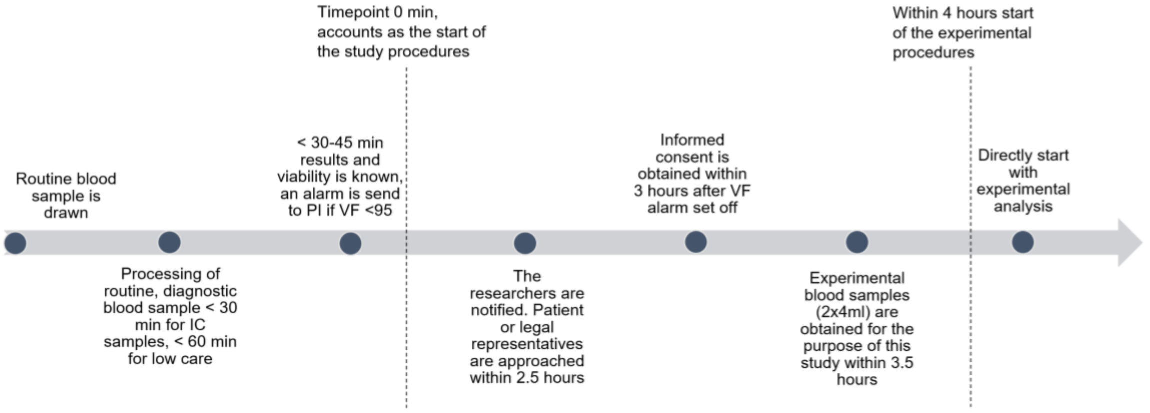


Figure 3. Time line for study and experimental processes

All routine diagnostic patient samples which are not processed within the time frame as stated in figure 3 will be excluded, because PI positivity is likely to be the result of prolonged bench time as explained below,

Leukocyte viability is reported by the hematology analyzer as the VF. The VF is the fraction of viable leukocytes within the total population of leukocytes. Viable leukocytes are PI-negative. To determine PI-positivity, leukocyte red-fluorescence is measured in the FL3-channel of the hematology analyzer. This measurement is routinely performed as part of the complete blood count measurement to distinguish older blood samples from fresh samples. Older blood samples contain more PI-positive leukocytes resulting in a lower VF. A VF of ≤ 0.95 is considered as the optimal cut-off value to differentiate between fresh and old samples.[7] The reliability and validity of all laboratory results are monitored with built-in quality flags, daily quality control samples and external quality assessment schemes.

*Experimental blood sampling* – When a VF ≤ 0.95 is detected in a fresh sample (as is normally the case within our hospital), additional blood samples will be collected after informed consent from the patient or representatives is obtained. In this case, VF ≤ 0.95 is not due to a prolonged bench time. The blood sampling will be executed at a single time point, as soon as possible, but no more than 3.5 hours after the alarm is set off. Two 4 milliliter (8ml total) sodium heparin blood containers are obtained to perform PI/Annexin

staining and the functional assays. After these assays, the blood samples will be destroyed.

It is key to obtain experimental blood samples within the period stated above, because of proposed short halftime of these neutrophils in the peripheral circulation. To make sure we get the blood samples that are most representative for the flagged routine sample, blood has to be drawn within the 3.5 hours. This way we can verify the findings in the routine sample and analyze the cells in the experimental sample.

*Annexin/Propidium iodide(PI) double staining (2 milliliter of 4 milliliter, blood container 1)* – Part of blood container 1 is used to retest neutrophil viability. Red blood cells are lyzed in isotonic ice-cold $NH_4Cl$ solution. Leukocytes are washed once and then re-suspended in PBS -/- to a concentration of approximately 2-4 x $10^6$ cells/ml. After which the cells are incubated consecutively with Annexin V binding buffer, Annexin V-Alexa Fluor 488 (Molecular Probes), and PI staining (Sigma).[5,8] The downside of Annexin V/PI staining is the occurrence of high false positive events because of PI staining of RNA in the cytoplasmic compartment. To limit this phenomenon the stained cells are treated with RNase prior to examination.[5] The number of apoptotic and necrotic neutrophils will be measured using a non-diagnostic flow cytometer. This will be expressed in viability and permits us to confirm or contest the VF measured in the routine blood sample with PI single stain. Samples with a high mean PI (>85) will be compared to samples with a normal mean PI (< 85). PI-positive neutrophils will be FACS-sorted from PI-negative neutrophils and used for fluorescence microscopy to determine the position of the PI stains (nuclear, extranuclear or extracellular).

*Neutrophil phagocytosis (1 milliliter of 4 milliliter, blood container 2)* – Part of blood container 2 is used for testing the phagocytic capacity of the neutrophils. The phagocytosis assay is performed in whole blood. The concentration of neutrophils is measured. The different bacteria (S. Aureus, E. Coli, H. Influenza, P. Aeruginosa) are constructed with promoter GFP and added to the whole blood sample with an MOI 1 -10. The whole blood with bacteria is shaken at 37 ℃ for 40 minutes. After 40 minutes the sample is put on ice and the leukocytes are stained with CD45 for recognition during analysis. Using flow cytometry leukocytes (CD45+), bacteria-associated neutrophils (CD45+/GFP+) can be identified. This assay will show the phagocytic capacity of the neutrophils in whole blood by comparing GFP+/- ratios of subjects to healthy controls. Also, samples with a high mean PI (>85) will be compared to samples with a normal mean PI (< 85).

*Neutrophil receptor expression and responsiveness (1 milliliter of 4 milliliter blood container 2)* – Part of blood container 2 is used for determination of the responsiveness to

the priming stimulus fMLP. The tube is placed in a Aquios load-and-go flow cytometer (Beckman Coulter). This flow cytometer is able to mix, pierce the cap, prepare and analyze the sample. Phage antibody A27 (previously described by our laboratory[9]) as well as other common neutrophil markers (e.g. CD16/CD11b/CD66b/ CD181/CD182/CD49d/CD62L) and blood are automatically pipetted in a microplate either containing or lacking the bacterial stimulus f-MLP. Samples with a high mean PI (>85) will be compared to samples with a normal mean PI (< 85). Via this assay we are able to investigate the differences in priming of neutrophils.

*Clinical data* – Data concerning age, gender, diagnosis during admission, type of surgery and inflammatory complications will be obtained from the patient administration system.

### 5.3 Withdrawal of individual subjects

Subjects can leave the study at any time for any reason if they wish to do so without any consequences. The investigator can decide to withdraw a subject from the study for urgent medical reasons. Furthermore, if a patient was included during unconsciousness via proxy consent and the patient progresses from a legally incapable status to a conscious and competent status we will inform the subject of the study and ask for informed consent from herself/himself. In case of refusal all prior obtained samples will be destroyed and data will be excluded. In case of signs of resistance of a patient with a legally incapacitated status the researchers will act according to the codes of conduct provided by the CCMO (http://www.ccmo.nl/nl/gedragscodes, chapter: Codes of conduct)

### 5.4 Replacement of individual subjects after withdrawal

After withdrawal of an included patient, the patient will be replaced by a consecutive patient with a VF ≤ 0.95 patient.

## 6. SAFETY REPORTING

### 6.1 Temporary halt for reasons of subject safety

In accordance to section 10, subsection 4, of the WMO, the sponsor will suspend the study if there is sufficient ground that continuation of the study will jeopardise subject health or safety. The sponsor will notify the accredited METC without undue delay of a temporary halt including the reason for such an action. The study will be suspended pending a further positive decision by the accredited METC. The investigator will take care that all subjects are kept informed.

## 6.2 AEs, SAEs

### 6.2.1 Adverse events

Adverse events (AE's) are defined as any undesirable experience occurring to a subject during the study, whether or not considered related to the experimental intervention. The AE's reported spontaneously by the subject or observed by the investigator or his staff, will be recorded during hospitalization if related to the venipuncture or blood drawing from a catheter.

### 6.2.2 Serious adverse events

A serious adverse event (SAE) is any untoward medical occurrence or effect that at any dose:

- results in death;
- is life threatening (at the time of the event);
- requires hospitalization or prolongation of existing inpatients' hospitalization;
- results in persistent or significant disability or incapacity;
- is a congenital anomaly or birth defect;
- Any other important medical event that may not result in death, be life threatening, or require hospitalization, may be considered a serious adverse experience when, based upon appropriate medical judgment, the event may jeopardize the subject or may require an intervention to prevent one of the outcomes listed above.

SAE's are common in the patient group enrolled in this study. We distinguish complications due to operations and adverse events due to the critical illness from other adverse events. The SAE's that will be reported are those which are related to the intervention, in this case either blood drawing via venipuncture or from the arterial catheter. The sponsor will report the SAEs through the web portal *ToetsingOnline* to the accredited METC that approved the protocol, within 15 days after the sponsor has first knowledge of the serious adverse reactions. SAEs listed below that result in death or are life threatening should be reported expedited. The expedited reporting will occur not later than 7 days after the responsible investigator has first knowledge of the SAE. This is for a preliminary report with another 8 days for completion of the report.

List of SAEs which will be reported:

- Line infection (arterial catheter, central venous line)
- Hypotension due to prolonged bleeding following venipuncture

### 6.3 Follow-up of adverse events

All AEs related to the study will be followed until they have abated, or until a stable situation has been reached. Depending on the event, follow up may require additional tests or medical procedures as indicated, and/or referral to the general physician or a medical specialist. SAEs related to the study need to be followed till end of study within the Netherlands, as defined in the protocol.

### 6.4 Data Safety Monitoring Board (DSMB) / Safety Committee

*Not applicable*

## 7. STATISTICAL ANALYSIS

The data will be analyzed using the software programs Excel, SPSS and Graphpad. The number of true positive events of decreased leukocyte viability will be determined for patients with a mean PI > 0.85 and patients with a mean PI < 0.85. Analysis of differences in true positive events between these groups will be performed with the use of a Fisher's exact test. Functionality assays (neutrophil phagocytosis and responsiveness) will be compared between patients with a VF ≤ 0.95 and healthy control patients. For this analysis the Unpaired T-test (in case of Gaussian distribution) or Mann Whitney U test (in case of non-normality) will be used. A subanalysis of neutrophil functionality will be performed between patients with a mean PI < 85 and patients with a mean PI > 85. Differences between these groups will be analyzed with the use of an Unpaired T-test or Mann Whitney U test depending on the data distribution.

### 7.1 Primary study parameter

The primary study parameter is neutrophil cell death: numbers, percentages and Fisher's exact

### 7.2 Secondary study parameters

- Position of DNA staining: numbers and percentages
- Neutrophil phagocytosis: unpaired T-test or Mann Whitney U test
- Neutrophil receptor expression: numbers and percentages

- Neutrophil responsiveness to fMLP: unpaired T-test or Mann Whitney U test

# 8. ETHICAL CONSIDERATIONS

## 8.1 Regulation statement

The study will be conducted according to the principles of the Declaration of Helsinki (64th WMA General Assembly, Fortaleza, Brazil, October 2013) and in accordance with the codes of conduct provided by the CCMO (http://www.ccmo.nl/nl/gedragscodes, chapter: Codes of conduct).

## 8.2 Recruitment and consent

Since neutrophil characteristics can rapidly change, it is essential to minimize the time between detection of a VF ≤ 0.95 and blood withdrawal. The PI, who is the main physician or part of the team of physicians treating this patient, receives a notification of the decreased VF. A notification without patient details will be send to the executive researcher. The PI will notify the patient and ask the patient if one of the executive researchers may inform the patient about the research and ask for informed consent. The executive researcher will approach the patient within 2.5 hours after the alarm has set off. Informed consent will be obtained as soon as possible, but no more than 3 hours after the alarm has set off. Thus, the patient has 30 minutes to consider informed consent. In case that a patient has a viability factor ≤ 0.95 for several days, another blood sample will be drawn after permission of the patient or his/her legally authorized representative. If the patient is incapacitated due to his or her injury or disease severity, we will attempt to obtain proxy consent. Proxy consent is obtained from the legally authorized representative, who is approached within 3 hours after the alarm is set off. The researcher will explain the study protocol after which the legally authorized representative has 30 minutes to consider informed consent, so that blood samples are obtained within 3.5 hours after the alarm is set off. When proxy consent was provided by the legally authorized representative during unconsciousness and the patient progresses from a legally incapable status to a conscious and competent status we will inform the subject of the study and ask for informed consent from herself/himself. No blood samples are taken without informed consent.

## 8.3 Objection by minors or incapacitated subjects

Not applicable.

### 8.4 Benefits and risks assessment, group relatedness

*Risk assessment* - A total of two 4 milliliter blood containers will be collected from the patients. Sampling this amount of blood will have a negligible influence on total blood volume and on the risk of developing anemia.[10] In total, one venipuncture is required to obtain the blood samples and if the patient has an arterial catheter in situ, no punctures are required.

*Benefit assessment* – A decreased viability of leukocytes was found in critically ill patients in previous studies with a fatal outcome. Not much is known about neutrophil viability in critically ill patients and the viability factor is not a commonly used laboratory parameter. A decrease in neutrophil viability might influence the antimicrobial response capacity and a decreased antimicrobial response capacity might lead to HAI. This study aims to elucidate the significance of a decreased VF and its relation with other signs of cell death. We hope to gain insights in the relation between VF and neutrophil functionality. This information is needed to get a better understanding of the neutrophil kinetics in critically ill surgical patients and its role in the pathogenesis of HAI.

### 8.5 Compensation for injury

The sponsor/investigator has a liability insurance which is in accordance with article 7 of the WMO.

The sponsor (also) has an insurance which is in accordance with the legal requirements in the Netherlands (Article 7 WMO). This insurance provides cover for damage to research subjects through injury or death caused by the study.
The insurance applies to the damage that becomes apparent during the study or within 4 years after the end of the study.

### 8.6 Incentives

Not applicable

## 9. ADMINISTRATIVE ASPECTS, MONITORING AND PUBLICATION

### 9.1 Handling and storage of data and documents

Data are handled confidentially. An individual subject will be connected to a subject identification code, not based on the patient's initials and birth-date but on number of

participation. The key to this code will be safe guarded by the executive researcher drs. L. Hesselink, and the principal investigator. The data will be saved on computers in the UMC Utrecht or on an encrypted USB drive. The handling of personal data will comply with the Dutch Personal Data Protection Act (*De wet Bescherming Persoonsgegevens*). The primary and secondary study endpoints described under ‘methods’ on page 12 will be documented by an independent researcher. Only the principal investigator (dr. Hietbrink) and the executive researchers drs. L. Hesselink and drs. R. Spijkerman are able to obtain data.

## 9.2 Monitoring and Quality Assurance

### 9.2.1 General

The purpose of the monitoring of clinical studies is to verify:

a. The rights and welfare of the subjects/patients are protected.
b. The study data reported is correct and complete and is verifiable in source documents.
c. The conduct of the study is consistent with the by METC approved protocol, Good Clinical Practice and the relevant legal requirements.

### 9.2.2 Monitoring plan

*Risk stratification*

Monitoring will take place according to the guidelines provided by the ‘Nederlandse Federatie van Universitaire Medische Centra’.[11] Following these guidelines this study is estimated by the PI as ‘negligible risk’ and monitoring will be performed as depicted in their regulations (Minimal monitoring). A monitoring plan is included in the next paragraphs. The monitoring plan and guidelines are also displayed in table 1 (page 21)

*Implementation of monitoring*

The on-site monitoring will be performed by an independent qualified monitor, provided by the Monitoring Pool of the division of Surgical Specialties of the UMC Utrecht.

*Frequency monitoring*

One regular monitoring visit per year is required. Since the duration of this study will be less than one year, there will be one visit only. Prior to the study an “initiation visit”

will be planned and at the end of the study a “close-out visit” will be planned. The close-out will be combined with the annual regular visit.

*Monitoring*

Monitoring is further described in file “K6. Monitoringplan versie 1 dd 31-05-2017”.

### 9.3 Amendments

Amendments are changes made to the research after a favorable opinion by the accredited METC has been given. All amendments will be notified to the METC that gave a favorable opinion.

### 9.4 Annual progress report

The sponsor/investigator will submit a summary of the progress of the trial to the accredited METC once a year. Information will be provided on the date of inclusion of the first subject, numbers of subjects included and numbers of subjects that have completed the trial, serious adverse events, other problems, and amendments.

### 9.5 Temporary halt and (prematurely) end of study report

The investigator/sponsor will notify the accredited METC of the end of the study within a period of 8 weeks. The end of the study is defined as the last patient’s last blood drawing.

The sponsor will notify the METC immediately of a temporary halt of the study, including the reason of such an action.

In case the study is ended prematurely, the sponsor will notify the accredited METC within 15 days, including the reasons for the premature termination.

Within one year after the end of the study, the investigator/sponsor will submit a final study report with the results of the study, including any publications/abstracts of the study, to the accredited METC.

### 9.6 Public disclosure and publication policy

The publication policy will be defined by the investigators themselves. All results will be published in international peer reviewed journals.

## 10. REFERENCES


1. Ishikawa H, Fukui T, Ino S, Sasaki H, Awano N. Influenza virus infection causes neutrophil dysfunction through reduced G-CSF production and an increased risk of secondary bacteria infection in the lung. *Virology*. 2016;499:23-29.
2. Kuijpers TW, Weening RS, Roos D. Clinical and laboratory work-up of patients with neutrophil shortage or dysfunction. *J Immunol Methods*. 1999;232(1-2):211-229.
3. Pietkiewicz S, Schmidt JH, Lavrik IN. Quantification of apoptosis and necroptosis at the single cell level by a combination of Imaging Flow Cytometry with classical Annexin V/propidium iodide staining. *J Immunol Methods*. 2015;423:99-103.
4. Hingorani R, Deng J, Elia J, McIntyre C, Mittar D. Detection of Apoptosis Using the BD Annexin V FITC Assay on the BD FACSVerseTM System. *BD Biosci*. 2011;August(August):1-12.
5. Rieger AM, Hall BE, Luong LT, Schang LM, Barreda DR. Conventional apoptosis assays using propidium iodide generate a significant number of false positives that prevent accurate assessment of cell death. *J Immunol Methods*. 2010;358(1-2):81-92.
6. Man WH, ten Berg M, Wilting I, Huisman A, Cahn W, Douma JW, den Breeijen H, Heerdink ER, Egberts T, van Solinge W. Fluorescence of neutrophil granulocytes as a biomarker for clozapine use. *Eur Neuropsychopharmacol*. 2013;23(11):1408-1413.
7. Huisman A, Stokwielder R, van Solinge WW. Mathematical correction of the invitro storage--related increase in erythrocyte mean cell volume of an automated hematology analyzer--the Cell-Dyn 4000. *Lab Hematol*. 2004;10(2):68-73.
8. Rieger AM, Nelson KL, Konowalchuk JD, Barreda DR. Modified annexin V/propidium iodide apoptosis assay for accurate assessment of cell death. *J Vis Exp*. 2011;(50):37-40.
9. Koenderman L, Kanters D, Maesen B, Raaijmakers J, Lammers JJ. Monitoring of neutrophil priming in whole blood by antibodies isolated from a synthetic phage antibody library Abstract : Neutrophil activation is a multistep process . In vitro activation of neutrophils with preactivation or priming with cytokines , chem. 2000.
10. Lyon AW, Chin AC, Slotsve G a, Lyon ME. Simulation of repetitive diagnostic blood loss and onset of iatrogenic anemia in critical care patients with a mathematical model. *Comput Biol Med*. 2012;1:1-7.
11. Nederlandse Federatie van Universitair Medische Centra. Kwaliteitsborging mensgebonden onderzoek 2.0. http://www.nfu.nl/img/pdf/NFU-12.6053_Kwaliteitsborging_mensgebonden_onderzoek_2.0.pdf. Published 2012.
