# Supplementary material for: Fragile neutrophils in surgical patients: A phenomenon associated with critical illness
Source: PLoS One. 2020 Aug 4;15(8):e0236596. doi: 10.1371/journal.pone.0236596 (PMC7402494; doi:10.1371/journal.pone.0236596)
Supplement: Attachment — Submitted filename: Response to reviewers.docx [file pone.0236596.s007.docx]

University Medical Center Utrecht

Heidelberglaan 100

3584 CX Utrecht

The Netherlands

June 14, 2020

PLOS ONE
1160 Battery Street
Koshland Building East, Suite 225
San Francisco, CA 94111
United States

Dear dr. Proost, dear dr. Heber, dear editorial board,

Thank you for your effort in reviewing our manuscript and we would like to thank the reviewers for their compliments. With their suggestions the manuscript gained clarity and improved substantially.

Attached you will find a list of the detailed changes made in the manuscript and a list of the responses to the comments of the reviewers. The changes are highlighted yellow in the manuscript.

We appreciate your time and effort and look forward to your response. If there are any remaining questions, we are happy to answer them.

Yours sincerely,

On behalf of the co-authors,

Lillian Hesselink, MD

Falco Hietbrink, MD/PhD

Department of Trauma surgery

University Medical Center Utrecht

**Reviewer #1**

Thank you for reviewing our manuscript and your compliments on this study. With your suggestions we added a few essential explanations and extra data to the manuscript.

Comments:

1) Please indicate the relative abundance of fragile neutrophils (percentage of total neutrophil subset) for patients that have these particular cells.

Thank you for this suggestion. We added this to table 1 (page 12). Also, Fig S2 gives insight in the number of non-viable leukocytes and the total number of leukocytes over time.

2) Data shown in figure 3 provide evidence for alterations in membrane marker expression of patient and control neutrophils. The observed differences might be related to differences in pathological state/inflammatory environment (e.g. alterations in plasma cytokine and chemokine levels that may cause neutrophil phenotypical and functional differences) rather than the presence of fragile neutrophils. Results from patients that do not have fragile neutrophils should be included in figures 3 and 4.

We completely agree that the differences in membrane marker expression can be related to differences in the inflammatory environment, such as alterations in plasma cytokines and chemokine levels, and that they are not the result of fragile neutrophils per se. We obtained these patient data, in combination with data from healthy controls, to get insight in the phenotypic characteristics of the complete neutrophil compartment of patients with fragile neutrophils. Although we were not able to include patients without fragile neutrophils in the prospective study, we have published extensively on the changes of neutrophils during acute systemic inflammation without the presence of fragile neutrophils. We have added these studies and considerations to the discussion section, paragraph 3 and 4.

3) Did the authors evaluate the plasma cytokine/chemokine levels in patients?

We did not evaluate plasma cytokine/chemokines levels in these patients. We agree that plasma cytokine/chemokines levels can give more insight into the inflammatory response. However, these mediators are divers and numerous and there is no real consensus which of these are important drivers of neutrophil inflammation. We chose to focus on the effector cell, the neutrophil, because neutrophils integrate these signals into a cellular response. As such, we hypothesized that neutrophil responses can be studied as “simple” biomarker of a complex inflammatory response. For this proof-of-principle study, we aimed to identify the presence of these fragile neutrophils and describe the phenomenon as such. Due to the very low incidence, inclusion and analysis is challenging. Nevertheless, in future studies, a more extensive investigation of other inflammatory mediators might be valuable. We have added these considerations to the discussion (line 306).

4) Is there a correlation between relative abundance of fragile neutrophils and alterations in membrane marker expression?

We analyzed the correlation between the white cell viability fraction (WVF) versus neutrophil marker expression in the prospectively included patients with fragile neutrophils (n = 9). There was no significant correlation between neutrophil marker expression and white cell viability fraction. This was added to the fourth paragraph of the discussion (line 271) and added as Supplementary Table S4.

We cannot rule out that no significant correlation was found due to the small study group we investigated, however, no marker stood out. Future studies should investigate neutrophil phenotypes in a larger cohort of patients with fragile neutrophils. We added this suggestion to the discussion, paragraph 6, line 306.

| **WVF vs.** | **Pearson correlation, R** | **P-value** |
| --- | --- | --- |
| CD35 | 0.432 | 0.246 |
| CD66b | 0.522 | 0.150 |
| CD64 | -0.038 | 0.922 |
| CBRM1/5 | 0.364 | 0.336 |
| CD11b | 0.521 | 0.150 |
| CD14 | -0.016 | 0.967 |
| CD16 | 0.278 | 0.469 |
| CD62L | -0.163 | 0.675 |
| LAIR1 | 0.184 | 0.636 |
| CD49d | 0.559 | 0.249 |

5) Data are too preliminary to make general conclusions concerning (absence of) alterations in neutrophil functionality in patients with fragile neutrophils. Assays were performed using whole blood samples or total leukocytes and only assess fMLF responsiveness and phagocytosis.

We agree that data on neutrophil functionality is still preliminary. Therefore we added the following sentences to the discussion:

Line 277: “These preliminary data on neutrophil functionality (responsiveness to fMLF and phagocytosis of bacteria) suggest that neutrophil function was not impaired in the prospectively included patients with a decreased WVF.”

Line 308: Due to this, data on neutrophil functionality in these patients is still preliminary. Future research should focus on other functional neutrophil tests to gain better insight in how the presence of fragile neutrophils influences overall neutrophil functionality.

6) Authors indicate that samples were processed within 3.5 hours, which can be tricky when working with neutrophils. Is there a correlation between time to processing and number of fragile neutrophils/phenotypical differences?

Blood sampling was always performed within 3.5 hours after the viability alarm went off (line 408). With that we meant that within 3.5 hours after the alarm, we obtained a new blood sample from the patient that was analyzed immediately. To check if the decreased white cell viability fraction (WVF) was not a result of processing time and to check if the fragile cells were still present in the peripheral blood of the patient, this new blood sample was again tested for the presence of fragile neutrophils by the hematology analyzer. We used this quick and fully automated hematology analyzer (Cell-dyn Sapphire) to minimize the time effect and increase the reproducibility of the test. This test was always performed within 15 minutes. Afterwards we started the sample processing, so that there was basically no time delay between the blood drawing and the start of the sample work-up. In all patients, fragile neutrophils were still present in the second blood sample.

To clarify this, we added this explanation to the methods section, line 410.

7) Did the authors ever detect fragile neutrophils in control samples?

Fragile neutrophils were never detected in control samples that were processed within 15 min. With a delay of 3-6h, no more than 2% fragile neutrophils were found in control samples.

8) Is there any evidence for morphological differences (e.g. differences in size or nuclear segmentation properties) of the fragile neutrophil subset?

If the necessary laboratory equipment was available, we also performed cell sorting in which we gated the PI+ (non-viable) cells and the PI- cells and made a cytospin. See the Figures below. Below are a few examples of the many cytospins we looked at. We analyzed these cytospins using the fluorescence microscope and found that the PI+ fragile neutrophils did not stay intact during sorting (black circles are examples in Figures 1 and 2). The cells that were not destroyed in de sort were mostly PI negative and presumably ended up in the PI+ sort because they stuck to PI+ neutrophils (red circles are examples in Figures 1 and 2). Hence, we were technically not able to find specific characteristics of the fragile neutrophil subset.

However, we did find some remarkable morphological characteristics in cytospins of patients with fragile neutrophils (in cells that stayed intact) (example is left Figure 1). Firstly, often these patients had many banded neutrophils and progenitor cells. Secondly, we found cytoplasmic vacuoles and toxic granulation in these neutrophils, which are all signs of severe infections/inflammation.^2,3^ These findings are in line with other findings that suggest severe inflammation in patients with fragile neutrophils, such as the changes in marker expression found in these patients and the number of severe infections found in these patients.

We added this to the discussion, line 259, and added two examples as supplement (S3 Fig).

**Figure 1**. Total neutrophils left vs PI+ neutrophils right

**Figure 2**. Total neutrophils left vs PI+ neutrophils right

9) Typo in Figure 3 panel A

Thank you for noticing this typo. We changed this in the Figure.

**Reviewer #2**

Thank you for your time and effort in reviewing the manuscript. Also thank you for your compliments regarding the importance of the paper.

Comments:

1) Figure 3: The authors mentioned in the text that expression of CBRM1/5 and CD14 were slightly higher in patient samples incubated fMLF. However, based on Figure 3J, CD14 expression is reduced in fMLF-stimulated neutrophils from patients compared to those from healthy controls.

Thank you for noticing this mistake. Indeed, CD14 expression was lower. We adjusted this in the text: “CBRM1/5 was slightly higher (p = 0.034) and CD14 was slightly lower (p = 0.023) than in healthy controls. “ (line 141).

**References**

^1^ Kabutomuri O. Toxic Granulation Neutrophils and C-Reactive Protein. Arch Intern Med. 2000;160(21):3326–3327.

^2^ McCall CE, Katayama I, Cotran RS, Finland M. Lysosomal and ultrastructural changes in human "toxic" neutrophils during bacterial infection. J Exp Med. 1969;129(2):267‐293. doi:10.1084/jem.129.2.267
