# Supplementary material for: Fragile neutrophils in surgical patients: A phenomenon associated with critical illness
Source: PLoS One. 2020 Aug 4;15(8):e0236596. doi: 10.1371/journal.pone.0236596 (PMC7402494; doi:10.1371/journal.pone.0236596)
Supplement: Attachment — Submitted filename: Response to reviewers.docx [file pone.0236596.s008.docx]

University Medical Center Utrecht

Heidelberglaan 100

3584 CX Utrecht

The Netherlands

June 14, 2020

PLOS ONE
1160 Battery Street
Koshland Building East, Suite 225
San Francisco, CA 94111
United States

Dear dr. Proost, dear dr. Heber, dear editorial board,

Thank you again for your effort in reviewing our manuscript and the responses. We think the insightful suggestions from the reviewers improved the manuscript. Attached you will find the response to the last question of reviewer 1 and the change we made in the manuscript. The changes are highlighted yellow in the manuscript.

We appreciate your time and effort and look forward to your response. If there are any remaining questions, we are happy to answer them.

Yours sincerely,

On behalf of the co-authors,

Lillian Hesselink, MD/PhD

Falco Hietbrink, MD/PhD

Department of Trauma surgery

University Medical Center Utrecht

**Reviewer #1**

Thank you again for reviewing our manuscript and reviewing the responses. Also, thank you for the insightful suggestions that improved the manuscript.

Comment: “*One last remark regarding the vacuoles (suppl. fig. S3): we usually observe these kind of 'vacuolated' neutrophils in patients that received corticosteroids. It may be interesting to check whether there is an association with therapy.”*

The prospectively included patients who had these vacuolated neutrophils did not receive corticosteroids before or during blood sampling. Because this was a relatively small group of patients, we additionally analyzed systemic corticosteroid use in the retrospectively included patients with fragile neutrophils. We found that 11 of the 74 patients (14.9%) were administered corticosteroids during admission. However, only 2 patients received corticosteroids during blood sampling. Moreover, no consistency in time-dependence could be observed between the start of corticosteroids and the presence of fragile neutrophils in the blood stream. Hence, it seems unlikely that the administration of corticosteroids was related to the presence of neutrophils with cytoplasmic vacuoles in patients with fragile neutrophils.

We added the following text to the **methods** section, line 536: “Also, it was investigated whether there was a relation between administration of corticosteroids and a decreased WVF, since corticosteroids are known to interfere with apoptosis of immune cells[1, 2]”

The following text was added to the **results** section, line 195: “Also, patients with fragile neutrophils more often received corticosteroids during admission (7.6% versus 14.9%, p = 0.027). However, only two (2.7%) patients received corticosteroids at the moment the viability alarm went off and no time-dependence was observed between the start of corticosteroids and the day the alarm went off.”

We added the following text to the **discussion**, line 265: “These characteristics are typical for severe inflammation and/or infection[3, 4] and can sometimes be seen after administration of corticosteroids[5]. However, the prospectively included patients who displayed these characteristics did not receive corticosteroids.”

**References**

1.	Schmidt S, Rainer J, Ploner C, et al (2004) Glucocorticoid-induced apoptosis and glucocorticoid resistance: Molecular mechanisms and clinical relevance. Cell Death Differ 11:S45–S55. https://doi.org/10.1038/sj.cdd.4401456

2.	Gruver-Yates A, Cidlowski J (2013) Tissue-Specific Actions of Glucocorticoids on Apoptosis: A Double-Edged Sword. Cells 2:202–223. https://doi.org/10.3390/cells2020202

3.	Kabutomkori O, Iwatani Y, Kanakura Y (2000) Toxic granulation neutrophils and C-reactive protein. Arch Intern Med 160:3326–7. https://doi.org/10.1001/archinte.160.21.3326-a

4.	McCall CE, Katayama I, Cotran RS, Finland M (1969) Lysosomal and ultrastructural changes in human “toxic” neutrophils during bacterial infection. J exp med 129:267–293

5.	Lee S, Khankhanian P, Mascarenhas JO (2015) Corticosteroid-induced morphological changes in cells of the myeloid lineage. Am J Hematol 90:679–680. https://doi.org/10.1002/ajh.23943
